# Fitness of *Escherichia coli* during Urinary Tract Infection Requires Gluconeogenesis and the TCA Cycle

**DOI:** 10.1371/journal.ppat.1000448

**Published:** 2009-05-29

**Authors:** Christopher J. Alteri, Sara N. Smith, Harry L. T. Mobley

**Affiliations:** Department of Microbiology and Immunology, University of Michigan Medical School, Ann Arbor, Michigan, United States of America; Yale University School of Medicine, United States of America

## Abstract

Microbial pathogenesis studies traditionally encompass dissection of virulence properties such as the bacterium's ability to elaborate toxins, adhere to and invade host cells, cause tissue damage, or otherwise disrupt normal host immune and cellular functions. In contrast, bacterial metabolism during infection has only been recently appreciated to contribute to persistence as much as their virulence properties. In this study, we used comparative proteomics to investigate the expression of uropathogenic *Escherichia coli* (UPEC) cytoplasmic proteins during growth in the urinary tract environment and systematic disruption of central metabolic pathways to better understand bacterial metabolism during infection. Using two-dimensional fluorescence difference in gel electrophoresis (2D-DIGE) and tandem mass spectrometry, it was found that UPEC differentially expresses 84 cytoplasmic proteins between growth in LB medium and growth in human urine (*P*<0.005). Proteins induced during growth in urine included those involved in the import of short peptides and enzymes required for the transport and catabolism of sialic acid, gluconate, and the pentose sugars xylose and arabinose. Proteins required for the biosynthesis of arginine and serine along with the enzyme agmatinase that is used to produce the polyamine putrescine were also up-regulated in urine. To complement these data, we constructed mutants in these genes and created mutants defective in each central metabolic pathway and tested the relative fitness of these UPEC mutants *in vivo* in an infection model. Import of peptides, gluconeogenesis, and the tricarboxylic acid cycle are required for *E. coli* fitness during urinary tract infection while glycolysis, both the non-oxidative and oxidative branches of the pentose phosphate pathway, and the Entner-Doudoroff pathway were dispensable *in vivo*. These findings suggest that peptides and amino acids are the primary carbon source for *E. coli* during infection of the urinary tract. Because anaplerosis, or using central pathways to replenish metabolic intermediates, is required for UPEC fitness *in vivo*, we propose that central metabolic pathways of bacteria could be considered critical components of virulence for pathogenic microbes.

## Author Summary

Bacteria that cause infections often have genes known as virulence factors that are required for bacteria to cause disease. Studying virulence factors such as toxins, adhesins, and secretion and iron-acquisition systems is a fundamental part of understanding infectious disease mechanisms. In contrast, little is known about the contribution of bacterial metabolism to infectious disease. This study shows that *E. coli*, which cause most urinary tract infections, utilize peptides as a preferred carbon source *in vivo* and requires some, but not all, of the central metabolic pathways to infect the urinary tract. Specifically, pathways that can be used to replenish metabolites, known as anaplerotic reactions, are important for uropathogenic *E. coli* infections. These findings help explain how metabolism can contribute to the ability of bacteria to cause a common infection.

## Introduction

Traditional studies of bacterial pathogenesis have focused on pathogen-specific virulence properties including toxins, adhesins, secretion, and iron acquisition systems, and mechanisms to avoid the innate and adaptive immune response. Examining bacterial metabolism during the course of an infection is also critical to further our understanding of pathogenesis and identifying potential targets for new antimicrobial agents. Infectious diseases represent a serious threat to global health because many bacteria that cause disease in humans such as *Staphylococcus aureus*, *Mycobacterium tuberculosis*, and *E. coli* are steadily developing resistance to many of the available treatments [1]–[3]. Since the introduction of antibiotics in the last century, the emergence of bacteria that resist these compounds has rapidly outpaced the discovery and development of new antimicrobial agents [4]. The need to understand bacterial physiology during infection of the host is critical for the development of new antimicrobials or antibiotics that will reduce their burden upon human health.

Among common infections, urinary tract infections (UTI) are the most frequently diagnosed urologic disease. The majority of UTIs are caused by *E. coli* and these uropathogenic *E. coli* (UPEC) infections place a significant financial burden on the healthcare system by generating annual costs in excess of two billion dollars [5],[6]. Because UTIs are a significant healthcare burden and *E. coli* is one of the best studied model organisms for studying metabolism, these traits can be exploited to understand and identify metabolic pathways that are required for the growth of the bacterium during infection of the host.

Despite being arguably the most studied organism, *E. coli* metabolism during colonization of the intestine has only recently been explored [7],[8]. Commensal *E. coli* acquires nutrients from intestinal mucus, a complex mixture of glycoconjugates, and subsequently expresses genes involved in the catabolism of N-acetylglucosamine, sialic acid, glucosamine, gluconate, arabinose and fucose [8],[9]. *E. coli* mutants in the Entner-Doudoroff and glycolytic central metabolic pathways have diminished colonization levels reflecting the importance of sugar acid catabolism [8]. These findings suggest that commensal *E. coli* uses multiple limiting sugars for growth in the intestine [8]. Together, this developing body of evidence supports the assertion that *E. coli* grows in the intestine using simple sugars released by the breakdown of complex polysaccharides by anaerobes [9],[10].

Much less is known about the metabolism of enteric pathogens during colonization of the gastrointestinal tract. Enterohemorrhagic *E. coli* (EHEC) O157∶H7 requires similar carbon metabolic pathways as do commensal strains, however, mutations in pathways that utilize galactose, hexuronates, mannose, and ribose resulted in colonization defects only for EHEC [9]. It was also found that multiple mutations in a single EHEC strain had an additive effect on colonization levels suggesting that this pathogen depends on the simultaneous metabolism of up to six sugars to support the colonization of the intestine [9]. When faced with limiting sugars due to consumption by other colonizing bacteria, EHEC may switch from glycolytic to gluconeogenic substrates to sustain growth in the intestine [11]. Synthesis and degradation of glycogen, an endogenous glucose polymer, plays an important role for EHEC and pathogenic *Salmonella* during colonization of the mouse intestine presumably by functioning as an internal carbon source during nutrient limitation [12]–[14]. Although it is not known which external carbon sources are used by *S. enterica* serovar Typhimurium during colonization it has been demonstrated that full virulence requires the conversion of succinate to fumarate in the tricarboxylic acid (TCA) cycle [15],[16]. These studies have contributed much to the understanding of the *in vivo* metabolic requirements of EHEC colonization; however, these studies were done in an animal model that is not suitable for studying pathogenesis because these animals do not exhibit signs of EHEC infection [9],[11],[13].

In contrast to the nutritionally diverse intestine, the urinary tract is a high-osmolarity, moderately oxygenated, iron-limited environment that contains mostly amino acids and small peptides [17],[18]. The available studies on UPEC metabolism during UTI has revealed that the ability to catabolize the amino acid D-serine in urine, which not only supports UPEC growth, appears important as a signaling mechanism to trigger virulence gene expression [19],[20]. Metabolism of nucleobases has been demonstrated to play a role for UPEC colonization of the urinary tract; signature-tagged mutagenesis screening identified a mutant in the dihydroorotate dehydrogenase gene *pyrD* that was outcompeted by wild-type UPEC *in vivo* [21] and in a separate transposon screen a gene involved in guanine biosynthesis, *guaA*, was identified and found to be attenuated during experimental UTI [22].

To better understand bacterial metabolism during infection, we used a combination of comparative proteomics and systematic disruption of central metabolism to identify pathways that are required for UPEC fitness *in vivo*. By examining the expression of UPEC cytoplasmic proteins during growth in human urine, we confirmed that *E. coli* is scavenging amino acids and peptides and found that disruption of peptide import in UPEC significantly compromised fitness during infection. Consistent with the notion that peptides are a key *in vivo* carbon source for UPEC, only mutations ablating gluconeogenesis and the TCA cycle demonstrated reduced fitness *in vivo* during experimental UTI. These findings represent the first study of pathogenic *E. coli* central metabolism in an infection model and further our understanding of the role of metabolism in bacterial pathogenesis.

## Results

### Proteomic profile for uropathogenic *E. coli* growing in urine

**Figure 1:**
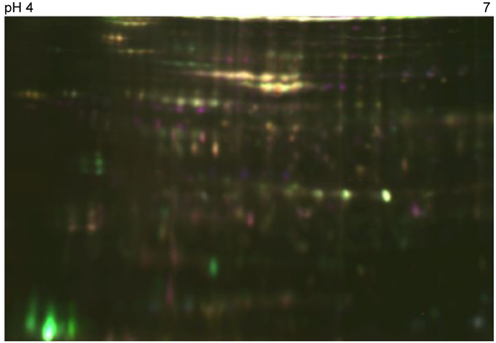
Fluorescence difference in gel electrophoresis (2D-DIGE) of UPEC cytoplasmic proteins during growth in urine. Soluble proteins (50 µg) from *E. coli* CFT073 cultured in urine were labeled with Cy3 (green), from CFT073 grown in LB with Cy5 (red), and the pooled internal standard representing an equal amount of urine and LB soluble proteins with Cy2 (blue). The labeled proteins (150 µg) were pooled and applied to a pH 4–7 IPG strip and second dimension 10% SDS-PAGE. Green spots indicate protein features induced in urine; red spots represent proteins induced in LB medium.

Culturing UPEC in human urine partially mimics the urinary tract environment and has proven to be a useful tool to identify bacterial genes and proteins involved in UTI [18], [22]–[24]. Because it is well established that urine is iron-limited and our previous studies clearly demonstrated that the majority of differentially expressed genes and proteins are involved in iron acquisition [18],[23], we determined the protein expression profile of *E. coli* CFT073 during growth in human urine and compared that with bacterial cells cultured in iron-limited LB medium to unmask proteins involved in processes other than iron metabolism. Using this strategy and 2D-DIGE it was possible to visualize 700 cytoplasmic protein spots, 84 of which were differentially expressed (*P*<0.05) between urine and iron-limited LB medium (Fig. 1). Of these, 56 were more highly expressed in human urine (green) than in iron-limited LB medium, while 28 demonstrated greater expression in iron-limited LB medium (red) than in urine (Fig. 1).

Proteins induced in human urine with >2-fold differences from expression levels in iron-limited LB medium were identified by tandem mass spectroscopy (Table 1). The results indicate that *E. coli* growing in urine are expressing proteins involved in the catabolism of pentose sugars; XylA (xylose isomerase), AraF (high-affinity arabinose-binding protein), and the non-oxidative pentose phosphate pathway enzyme TalA (transaldolase) were induced 5.25-, 2.02-, and 5.66-fold, respectively (*P*<0.001) (Table 1). Other proteins that were induced are the involved in metabolism of the sugar acids gluconate (UxuA, mannonate dehydratase), gluconolactone (YbhE, 6-phosphogluconolactonase), sialic acid (NanA, N-acetylneuraminate lyase), and fructose (FruB, fructose-specific IIA/FPr PTS system component). Multiple isoforms of the periplasmic dipeptide and oligopeptide substrate-binding proteins DppA and OppA were also induced (>2-fold, *P*<0.009) in urine confirming the notion that amino acids and small peptides are being acquired from this milieu (Table 1). Proteins involved in amino acid metabolism were also identified and include SerA (D-3-phosphoglycerate dehydrogenase) that is involved in serine biosynthesis and two enzymes in the arginine biosynthesis pathway, ArgG (argininosuccinate dehydrogenase) and SpeB (agmatinase) (Table 1). As expected, none of the proteins identified were involved in iron uptake or metabolism, although DppA has been reported to bind heme albeit with less affinity than dipeptide substrates [25].

**Table 1:** UPEC cytoplasmic proteins differentially expressed in human urine.

Name	ORF	Function	Fold-Change	*P*-Value
OmpF	c1071	outer membrane protein F precursor	7.84	2.50E-11
OmpF	c1071	outer membrane protein F precursor	5.97	2.33E-05
TalA	c2989	transaldolase	5.66	0.00021
XylA	c4385	xylose isomerase	5.25	6.90E-07
TpiA	c4871	triosephosphate isomerase	4.58	1.30E-07
SerA	c3494	D-3-phosphoglycerate dehydrogenase	4.44	3.40E-09
SpeB	c3522	agmantinase	4.06	3.90E-07
UxuA	c5402	mannonate dehydratase	3.76	7.20E-03
NanA	c3979	N-acetylneuraminate lyase subunit	3.64	4.50E-06
ArgG	c3929	argininosuccinate dehydrogenase	3.41	5.80E-03
FklB	c5306	peptidyl-prolyl cis trans isomerase	3.38	6.00E-04
NanA	c3979	N-acetylneuraminate lyase subunit	3.37	4.50E-06
AtpA	c4660	ATP synthase subunit A	3.34	6.30E-05
XylA	c4385	xylose isomerase	3.32	5.60E-05
NmpC	c1560	outer membrane protein NmpC precursor	3.3	6.10E-05
FruB	c2704	PTS system, fructose-specific IIA/FPr component	2.93	3.40E-06
RpoA	c4056	DNA-directed RNA polymerase	2.84	4.40E-04
GlyA	c3073	serine hydroxymethyl transferase	2.72	1.50E-10
LivK	c4248	leucine-specific binding protein	2.72	2.90E-08
FruB	c2704	PTS system, fructose-specific IIA/FPr component	2.71	3.20E-04
DppA	c4361	dipeptide substrate-binding protein	2.63	5.20E-04
SurA	c0066	peptidyl-prolyl cis trans isomerase	2.61	3.10E-07
YliJ	c0923	hypothetical GST protein	2.61	4.00E-04
HisJ	c2851	histidine-binding protein precursor	2.55	1.90E-04
ArgG	c3929	argininosuccinate dehydrogenase	2.41	2.60E-02
OppA	c1707	oligopeptide substrate-binding protein	2.39	7.80E-03
OppA	c1707	oligopeptide substrate-binding protein	2.34	2.10E-04
SerA	c3494	D-3-phosphoglycerate dehydrogenase	2.28	1.90E-05
YghU	c3726	hypothetical GST-like protein	2.27	1.10E-05
YbhE	c0844	6-phosphogluconolactonase	2.2	9.90E-03
SucC	c0805	succinyl-CoA synthetase beta chain	2.14	1.50E-04
GlpA	c2782	anaerobic glycerol-3-phosphate dehydrogenase	2.13	3.50E-07
XylA	c4385	xylose isomerase	2.11	1.30E-02
MalK	c5005	maltose/maltodextran ATP-binding	2.1	6.90E-03
DppA	c4361	dipeptide substrate-binding protein	2.09	8.40E-03
NmpC	c1560	outer membrane protein NmpC precursor	2.03	1.80E-03
AraF	c2314	L-arabinose-binding protein	2.02	2.80E-06
UxuA	c5402	mannonate dehydratase	1.94	8.30E-04
AsnS	c1072	asparaginyl-tRNA synthetase	1.9	1.20E-02
GlnH	c0896	glutamine-binding protein	1.68	1.40E-03
GroEL	c5227	chaperonin	−2.07	8.90E-08
GroEL	c5227	chaperonin	−2.07	7.10E-05
NusA	c3926	transcription elongation factor	−2.1	3.30E-02
BasR	c5118	transcription factor	−2.91	5.50E-03
HdeB	c4320	acid resistance protein precursor	−3.71	2.50E-04

Notably, there was an increase in abundance for two central metabolism enzymes, TalA, as mentioned above, and TpiA that was increased 4.58-fold (*P*<0.0001) in urine (Table 1). TalA, a non-oxidative pentose phosphate pathway enzyme, converts sedoheptulose-7-phosphate and glyceraldehyde-3-phosphate to erythrose-4-phosphate and fructose-6-phosphate. Due to the transfer of the glycolytic intermediate glyceraldehyde-3-phosphate by TalA, this enzyme is an important link between the pentose phosphate pathway and glycolysis [26]. TpiA is a glycolytic enzyme that catalyzes the reversible isomerization of glyceraldehyde-3-phosphate and dihydroxyacetone phosphate [27]. The induction of TalA and TpiA suggested that the coupling of the pentose phosphate pathway and glycolysis or gluconeogenesis via the transfer and isomerization of glyceraldehyde-3-phosphate may be an important route of carbon flux through these central pathways during the bacterium's growth in human urine.

### Contribution of genes induced in urine to UPEC fitness *in vivo*

To determine whether some proteins identified by 2D-DIGE are required for UPEC fitness during UTI, CFT073 mutants were constructed in the genes: *talA*, *xylA*, *tpiA*, *serA*, *speB*, *uxuA*, *nanA*, *argG*, *araF*, *dppA*, and *oppA*. For these studies, an experimental competition between each mutant strain and wild-type parental CFT073 was performed. Wild-type UPEC and the mutant strain were prepared in a 1∶1 ratio and transurethrally inoculated into the bladders of mice. The number of mutant (kanamycin-resistant) and wild-type (kanamycin-sensitive) bacteria recovered from the bladder and kidneys was determined by plating the tissue homogenates for CFU on both LB agar and LB agar containing kanamycin. Mutants containing defects in genes that affect fitness *in vivo* are out-competed by the wild-type strain when inoculated into the same animal. This was determined by comparing the ratio of colony forming units (CFU) of bacteria recovered from the infection to the ratio of bacteria contained within the inoculum to obtain a competitive index (CI). A CI>1 indicates the wild-type out-competes the mutant strain and a CI<1 indicates the wild-type is out-competed by the mutant. In these series of experimental infections, only mutants defective in peptide transport (Δ*dppA* and Δ*oppA*) were dramatically out-competed by wild-type UPEC *in vivo*, CI>50, *P*<0.005 for the bladder (Table 2). One additional mutant, Δ*tpiA*, that functions in both glycolysis and gluconeogenesis, was out-competed by wild-type in the kidneys at 48 hpi, CI = 2.54, *P* = 0.0206 (Table 2).

**Table 2:** *In vivo* fitness for select 2D-DIGE mutants.

	Bladder		Kidneys	
	CI^a^	*P*-Value^b^	CI^a^	*P*-Value^b^
*talA*	0.150	0.1282	0.660	0.3829
*xylA*	1.66E−02	0.0625	0.233	0.0649
*tpiA*	0.841	0.4050	**2.540**	**0.0206**
*serA*	5.310	0.4206	1.58	0.5476
*speB*	**0.140**	**0.0122**	2.248	0.3652
*uxuA*	0.397	0.0667	0.608	0.1750
*nanA*	0.659	0.1875	1.240	0.4075
*argG*	0.160	0.0625	1.970	0.3750
*araF*	0.854	0.4401	0.297	0.4507
*dppA*	**56.33**	**0.0020**	1.408	0.5625
*oppA*	**4.77E+02**	**0.0047**	**1.56E+02**	**0.0420**

a Competitive Index, determined by dividing the ratio of wild-type to mutant at 48 hpi by the ratio present in the inoculum. Significant CI>1 indicates mutant has a fitness defect.

b *P*-values determined by Wilcoxon matched pairs test. Significant *P*-values are bolded.

**Figure 2:**
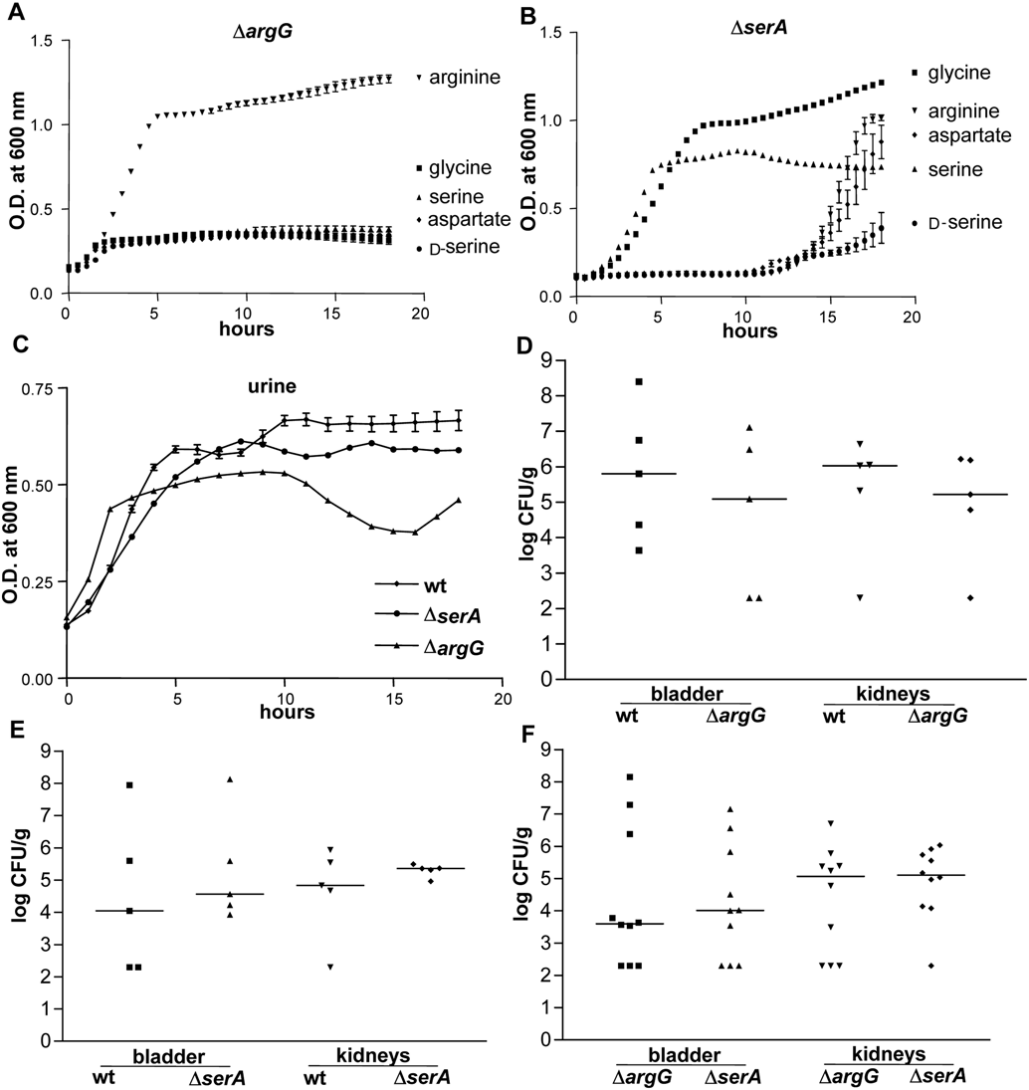
*In vivo* contribution of UPEC arginine and serine biosynthesis. Demonstration of auxotrophic phenotypes for (A) Δ*argG* and (B) Δ*serA* in MOPS defined medium containing 0.2% glucose and 10 mM of the indicated amino acid. (C) Growth in human urine. Growth curves represent the average measurement at each time point from triplicate experiments. Individual female mice were transurethrally inoculated with 2×10^8^ CFU of a 1∶1 mixture of wild-type and mutant bacteria. *In vivo* fitness at 48 h post infection (hpi) for UPEC mutants defective in (D) arginine and (E) serine biosynthesis. (F) *In vivo* competition between arginine and serine auxotrophy. At 48 hpi, bladders and kidneys were aseptically removed, homogenized, and plated on LB or LB containing kanamycin to determine viable counts of wild-type and mutant strains, respectively. Each dot represents the log CFU/g from an individual animal. Bars represent the median CFU/g, and the limit of detection is 200 CFU. Significant differences in colonization levels (*P*<0.05) were determined using a two-tailed Wilcoxon matched pairs test.

Despite the lack of attenuation *in vivo* for the many of the mutants, these results reveal a number of important findings. The agmatinase mutant Δ*speB* out-competed wild-type in the bladder at 48 hpi, CI = 0.14, *P* = 0.0122 (Table 2). Agmatinase is part of arginine metabolism and catalyzes the formation of the polyamine putrescine and urea from agmatine and H_2_O. This suggests that accumulation of agmatine or reduced production of urea and putrescine by the mutant may provide a modest advantage over wild-type UPEC during infection of the bladder. CFT073 Δ*argG* was unable to grow in MOPS defined medium unless supplemented with 10 mM arginine (Fig. 2A), validating the expected auxotrophic phenotype. Similarly, the Δ*serA* serine auxotroph required supplementation with either 10 mM serine or glycine in MOPS, D-serine was unable to rescue the *in vitro* growth defect (Fig. 2B). Lack of arginine or serine biosynthesis had little effect upon the ability of UPEC to grow logarithmically in human urine, although the Δ*argG* mutant consistently entered stationary phase at a lower cell density, with an O.D._600_ of 0.45±0.04 compared to 0.59±0.03 for wild-type (*P* = 0.051) (Fig. 2C). When tested for *in vivo* fitness, neither the Δ*argG* nor Δ*serA* strain were significantly out-competed by wild-type UPEC at 48 hpi (Fig. 2D, 2E, and Table 2). Additionally, there was no preference for serine over arginine or vice versa for UPEC colonization at 48 hpi. When the auxotrophic strains were co-inoculated into the same mice both mutants were recovered at similar levels (Fig. 2F). These data clearly demonstrate that there are sufficient concentrations of arginine, serine and/or glycine in the urinary tract to support growth of these auxotrophic strains.

**Figure 3:**
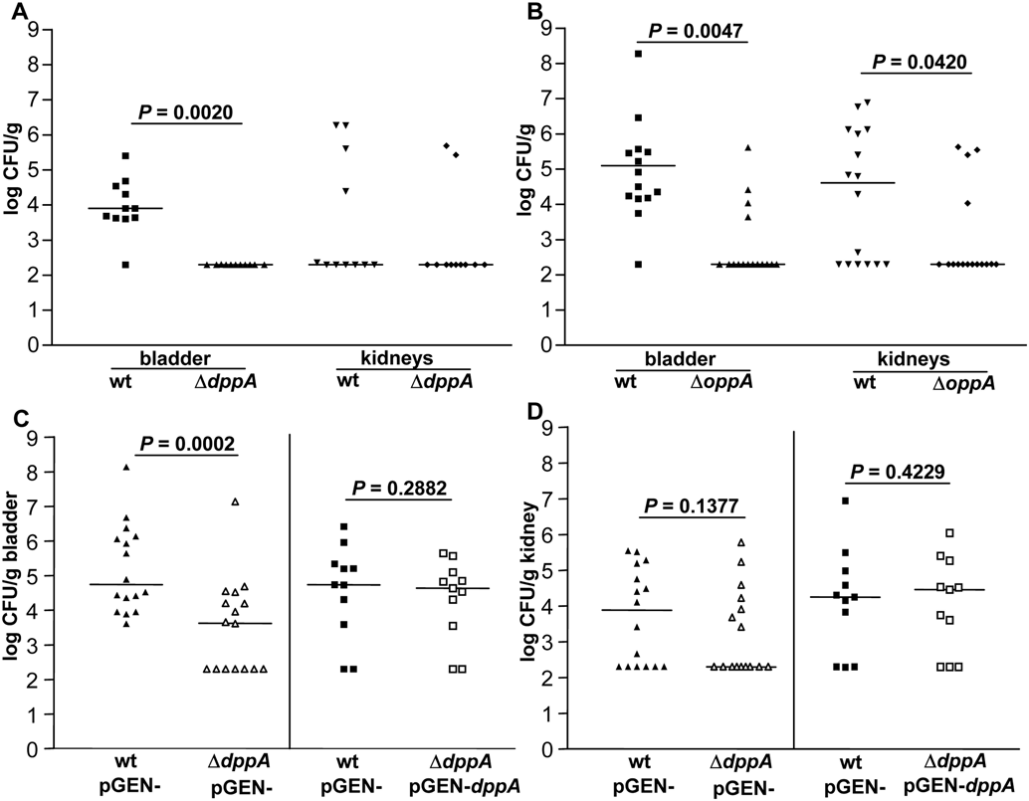
*In vivo* contribution of UPEC peptide substrate-binding proteins. Individual female mice were transurethrally inoculated with 2×10^8^ CFU of a 1∶1 mixture of wild-type and mutant bacteria. *In vivo* fitness at 48 hpi for UPEC mutants defective in import of dipeptides (A) Δ*dppA* or oligopeptides (B) Δ*oppA*. At 48 hpi, bladders and kidneys were aseptically removed, homogenized, and plated on LB or LB containing kanamycin to determine viable counts of wild-type and mutant strains, respectively. *In vivo* complementation of Δ*dppA* was performed by inoculating mice with a mixture of wild-type CFT073 containing pGEN empty vector and Δ*dppA* containing pGEN empty vector or pGEN-*dppA*. At 48 hpi, (C) bladders and (D) kidneys were aseptically removed, homogenized, and plated on LB with ampicillin or LB containing ampicillin and kanamycin to determine viable counts of wild-type (closed symbols) and mutant strains (open symbols), respectively. Each dot represents the log CFU/g from an individual animal. Bars represent the median CFU/g, and the limit of detection is 200 CFU. Significant differences in colonization levels (*P*<0.05) are indicated and were determined using a two-tailed Wilcoxon matched pairs test.

As mentioned, deletion of the genes encoding periplasmic peptide substrate-binding proteins, *dppA* and *oppA*, had the greatest impact on UPEC fitness *in vivo* of the CFT073 mutants in genes whose products were induced during growth in human urine (Table 2). The dipeptide transport mutant, Δ*dppA*, failed to maintain colonization in the bladder at 48 hpi, 11/11 bladders had undetectable levels (<200 CFU/g) for this mutant, while wild-type levels from the same bladders reached a median of 10^4^ CFU/g (*P* = 0.0020) (Fig. 3A). Because these mice had low levels of recoverable UPEC from the kidneys it was not possible to determine the contribution of dipeptide transport for kidney colonization. Import of oligopeptides via the OppA substrate-binding protein is also required for UPEC fitness *in vivo*. CFT073 Δ*oppA* was out-competed nearly 500∶1 wild-type∶mutant in the bladder (Table 2) with a 3-log reduction in the median CFU/g from bladder tissue at 48 hpi (*P* = 0.0047) (Fig. 3B). In these co-challenge infections, wild-type UPEC colonized 10/16 (62%) of kidneys, while Δ*oppA* was detectable in 4/16 (25%) of kidneys at 48 hpi. The ratio of wild-type∶mutant recovered from the kidneys at this time point was 156∶1 (Table 2) where wild-type UPEC had 3-logs greater CFU/g than Δ*oppA* (*P* = 0.0420) (Fig. 3B). Together, the *in vivo* fitness defect for CFT073 harboring a deletion of either *dppA* or *oppA* suggests that peptides may be an important carbon source for UPEC during urinary tract infection.

Previously, we have shown that the low copy pGEN plasmid is maintained in CFT073 in the absence of antibiotic pressure for up to 48 h [28]. Using this ampicillin resistant plasmid system, we cloned the entire *dppA* gene including 200 bp upstream from the predicted start site of translation and introduced the resulting construct, pGEN-*dppA*, into the CFT073 Δ*dppA* strain. To determine if it was possible to complement the Δ*dppA* defect *in vivo*, co-challenge infections were performed as described and modified to enumerate bacteria in tissue homogenates by plating on agar containing ampicillin (wild-type CFT073 harboring pGEN) or ampicillin and kanamycin (CFT073 Δ*dppA* containing pGEN or pGEN-*dppA*). The Δ*dppA* mutant containing empty vector (pGEN-) demonstrated the expected fitness defect in bladder colonization when co-inoculated with wild-type CFT073 (pGEN-) (*P* = 0.0002) while Δ*dppA* containing a wild-type copy of *dppA* (pGEN-*dppA*) restored colonization to wild-type levels in the bladder at 48 hpi (Fig. 3C). Although both mutant (pGEN-) and wild-type (pGEN-) demonstrated poor colonization in the kidneys of these animals, complementation of Δ*dppA* (pGEN-*dppA*) resulted in a 2-log increase in median kidney CFU/g at 48 hpi (Fig. 3D).

### Fitness of UPEC central carbon metabolism mutants during UTI

**Figure 4:**
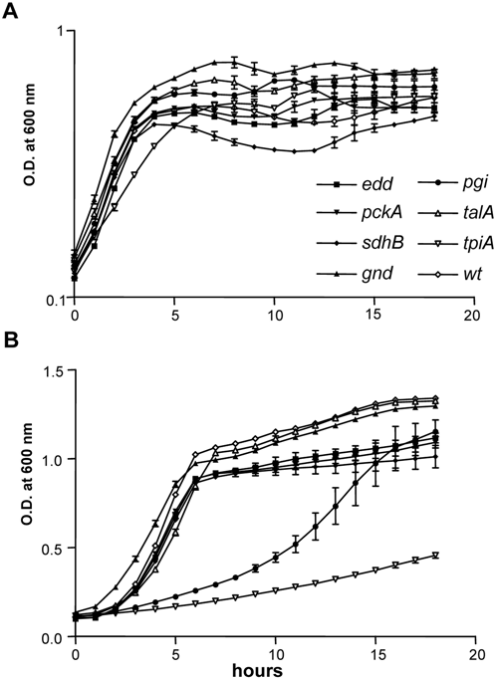
*In vitro* growth of UPEC central metabolism mutants. Optical density of wild-type UPEC and central metabolism mutants during growth in (A) pooled and sterilized human urine from 8–10 donors and in (B) MOPS defined medium containing 0.2% glucose as the sole carbon source. Growth curves represent the average measurement at each time point from triplicate experiments.

The requirement for peptide transport for UPEC fitness during infection implicates peptides as an important carbon source *in vivo*. This predicts that certain central metabolism pathways that operate during catabolism of amino acids or peptides may be more important for *in vivo* growth of UPEC than pathways that function primarily to catabolize sugars. To test the role of central metabolic pathways during an actual infection mutants were constructed in UPEC strain CFT073 to produce defects in glycolysis (*pgi*, phosphoglucose isomerase and *tpiA*, triosephosphate isomerase) [29], the Entner-Doudoroff pathway (*edd*, 6-phosphogluconate dehydratase) [10], the oxidative branch (*gnd*, 6-phosphogluconate dehydrogenase) and the non-oxidative branch (*talA*, transaldolase) of the pentose phosphate pathway [26], gluconeogenesis (*pckA*, phosphoenolpyruvate carboxykinase) [30], and the TCA cycle (*sdhB*, succinate dehydrogenase) [31]. The *in vitro* growth of these central metabolism mutants were examined and compared to wild-type UPEC during culture in human urine, LB medium, and MOPS defined medium containing 0.02% glucose. All of the central metabolism mutants produced similar logarithmic growth as wild-type when cultured in human urine (Fig. 4A) and LB medium (data not shown) under defined inoculation conditions. As expected, only mutants with defects in glycolysis demonstrated diminished growth in MOPS medium containing glucose as the sole carbon source (Fig. 4B). The Δ*pgi* strain produced an extended lag phase of 5.5±1.1 h compared with wild-type (*P* = 0.001) and Δ*tpiA* failed to reach exponential phase after 18 h (Fig. 4B). These data and the indistinguishable growth of the glycolysis mutants from wild-type in urine supported the proteomics data and indicated that UPEC growing in urine utilizes carbon sources other than glucose.

**Figure 5:**
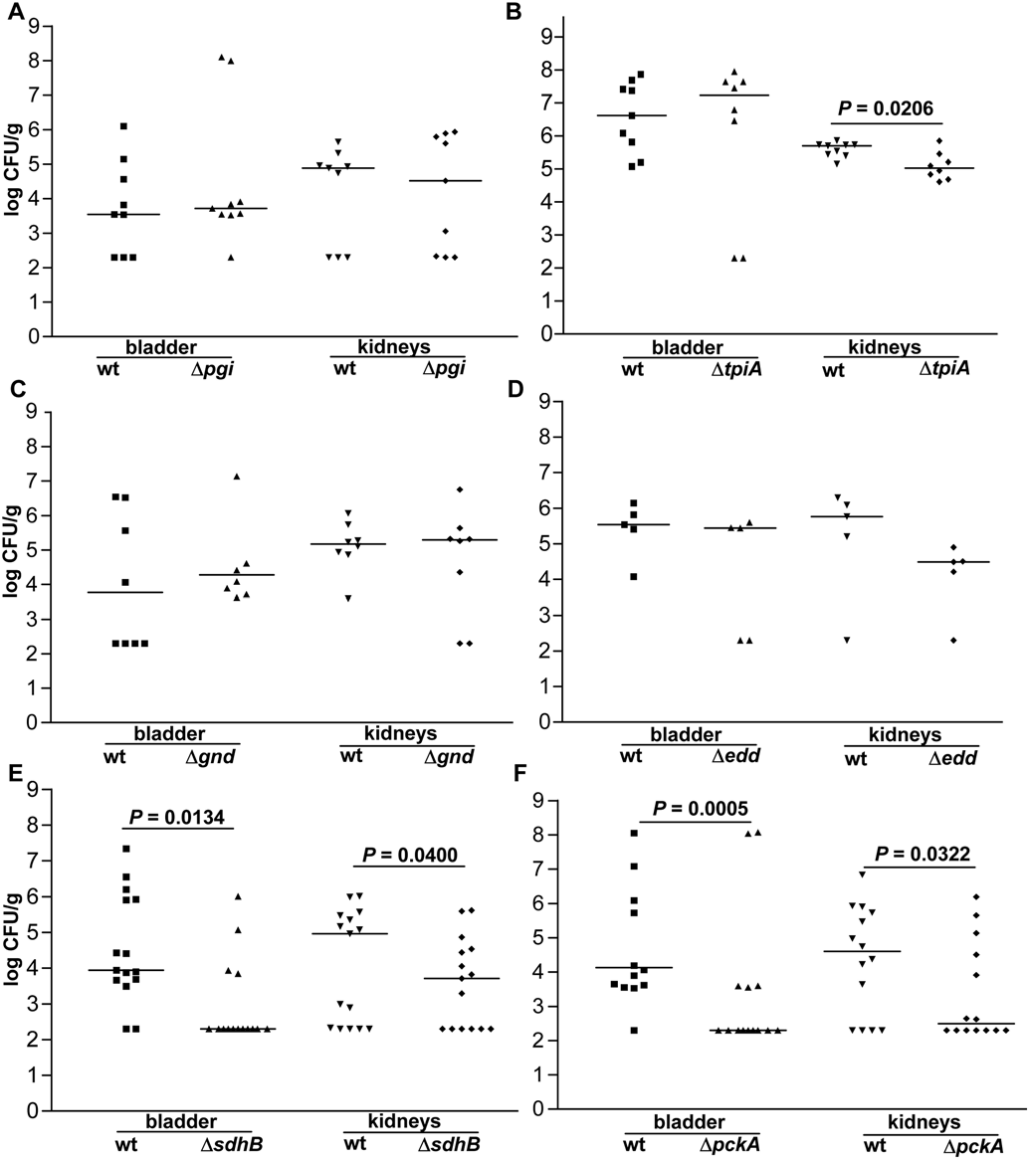
*In vivo* fitness of UPEC central metabolism mutants. Individual female mice were transurethrally inoculated with 2×10^8^ CFU of a 1∶1 mixture of wild-type and mutant bacteria. *In vivo* fitness at 48 hpi for UPEC mutants defective in: (A,B) glycolysis, (C) pentose phosphate pathway, (D) Entner-Doudoroff pathway, (E) TCA cycle, and (F) gluconeogenesis. At 48 hpi, bladders and kidneys were aseptically removed, homogenized, and plated on LB or LB containing kanamycin to determine viable counts of wild-type and mutant strains, respectively. Each dot represents the log CFU/g from an individual animal. Bars represent the median CFU/g, and the limit of detection is 200 CFU. Significant differences in colonization levels (*P*<0.05) are indicated and were determined using a two-tailed Wilcoxon matched pairs test.

To determine the role for central metabolism during *E. coli* infection of the urinary tract, the ascending model of murine UTI was used as described above to measure the impact that a lesion in central metabolism has upon the relative fitness of the strain *in vivo*. Mutants with defects in glycolysis had levels of colonization in the bladder at 48 hpi similar to wild-type (*P*>0.400) (Fig. 5A and 5B). In the kidneys, Δ*pgi* CFU/g were comparable to wild-type (Fig. 5A), while Δ*tpiA* demonstrated a 10-fold reduction in the median CFU/g (*P* = 0.0206) (Fig. 5B). The pentose phosphate pathway mutants, Δ*gnd* (Fig. 5C) and Δ*talA* (Table 2), were not significantly out-competed by wild-type *in vivo*. The mutant with a defect in the Entner-Doudoroff pathway (Δ*edd*) also was not impaired in the ability to infect both the bladder and kidneys as indicated by its similar colonization to wild-type at 48 hpi (Fig. 5D). UPEC *in vivo* fitness was significantly reduced in the TCA cycle mutant Δ*sdhB*, this mutation resulted in a 50-fold reduction in median CFU/g in the bladder (*P* = 0.0134) and a 1.5-log decrease in kidney CFU at 48 hpi (*P* = 0.0400) (Fig. 5E). This defect in the TCA cycle impacted fitness to a greater extent in the bladder, where 11/15 (73%) of mice had undetectable levels of mutant bacteria, than in the kidneys where 6/15 (40%) mice had undetectable counts (Fig. 5E). The gluconeogenesis mutant, Δ*pckA* had a 2-log reduction in median CFU/g in both the bladder (*P* = 0.0005) and kidneys (*P* = 0.0322) and half of the mice (7/14) displayed undetectable levels of Δ*pckA* at 48 hpi (Fig. 5F).

**Figure 6:**
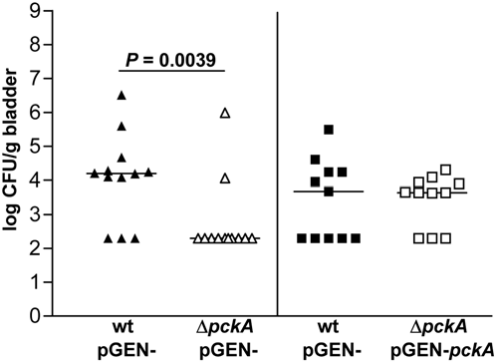
*In vivo* complementation of UPEC Δ*pckA*. Individual female mice were transurethrally inoculated with 2×10^8^ CFU of a 1∶1 mixture of wild-type CFT073 containing pGEN empty vector and Δ*pckA* containing pGEN empty vector or pGEN-*pckA*. At 48 hpi, bladders were aseptically removed, homogenized, and plated on LB with ampicillin or LB containing ampicillin and kanamycin to determine viable counts of wild-type (closed symbols) and mutant strains (open symbols), respectively. Bars represent the median CFU/g, and the limit of detection is 200 CFU. Significant differences in colonization levels (*P*<0.05) are indicated and were determined using a two-tailed Wilcoxon matched pairs test.

To verify that this mutation is non-polar as expected and the defect in colonization is not due to a secondary mutation, *in vivo* complementation experiments were conducted. The Δ*pckA* mutant with the pGEN empty vector demonstrated a 2-log reduction in CFU/g at 48 hpi (*P* = 0.0039) in the bladder when co-inoculated into mice with wild-type UPEC containing pGEN (Fig. 6). When CFT073 Δ*pckA* (pGEN-*pckA*) were co-inoculated with CFT073 (pGEN-) there was no significant difference in bladder CFU/g at 48 hpi between the strains (Fig. 6). Thus, by re-introducing the *pckA* gene into the mutant it was possible to complement the Δ*pckA* defect in bladder colonization at 48 hpi.

The *in vitro* growth and *in vivo* fitness for the UPEC central metabolism mutants is summarized in Table 3. As expected, only mutations in glycolysis had a negative effect on growth in defined medium with glucose. Only gluconeogenesis or TCA cycle mutants demonstrated reduced persistence at 48 hpi in both the bladder and kidneys (Table 3). Non-oxidative and oxidative pentose phosphate pathway and Entner-Doudoroff pathway mutants did not demonstrate any colonization defect and of the glycolytic mutants only the triosephosphate isomerase deletion had a measurable defect in the kidneys but not in the bladder (Table 3). Together, the fitness defect for the peptide transport mutants and these data indicate UPEC could be using amino acids as the primary carbon source during infection. Surprisingly, there was no correlation between the ability of the central metabolism mutants to grow in human urine *ex vivo* and grow in the urinary tract *in vivo*.

**Table 3:** Growth of central metabolism mutants *in vitro* and *in vivo*.

Mutant	Pathway	*In Vitro* Growth			*In Vivo*
		LB	Urine	Glucose	Colonization Defect
*edd*	Entner-Doudoroff	+	+	+	None
*gnd*	Pentose phosphate	+	+	+	None
*pckA*	Gluconeogenesis	+	+	+	Bladder, Kidneys
*pgi*	Glycolysis	+	+	−	None
*sdhB*	TCA cycle	+	+	+	Bladder, Kidneys
*talA*	Pentose phosphate	+	+	+	None
*tpiA*	Glycolysis	+	+	−	Kidneys

## Discussion

Bacterial pathogenesis traditionally involves studying virulence traits involved in the production of toxins and effectors, iron acquisition, adherence, invasion, and immune system avoidance. Although many paradigms exist that describe mechanisms of pathogenesis, the contribution of microbial metabolism to bacterial virulence during an infection is less understood. Much work has been done studying *E. coli* as model organism for characterizing individual central metabolism pathways and enzymes [10], [27], [32]–[38]. We have shown here that central metabolism studies in *E. coli* can be extended to investigate the contribution of central pathways to bacterial pathogenesis using a virulent uropathogenic *E. coli* strain and a well-established animal model of UTI. It is known that commensal *E. coli* require the Entner-Doudoroff pathway and glycolysis for colonization *in vivo*; while the TCA cycle, pentose phosphate pathway, and gluconeogenesis are dispensable in the intestine [8]. In contrast, we have shown that during *E. coli* infection of the urinary tract, the pathways required for commensal colonization are dispensable while the TCA cycle and gluconeogenesis are necessary for UPEC fitness *in vivo*. Adaptation to distinct host environments has been previously shown to involve shared traits between commensal and pathogenic strains [39],[40]. Because commensal *E. coli* are an important natural component of the intestine one concern faced when developing antimicrobials that target pathogenic strains is how to avoid eradicating commensal bacteria. Thus, these findings highlight important differences between commensal and pathogenic *E. coli* that could be exploited for the development of antimicrobials that target these pathways in this pathogen during infections that may not affect commensal strains. Interestingly, in addition to UPEC, gluconeogenesis is required for virulence in microbes that represent an array of pathogenic lifestyles, from intracellular bacteria and parasites [41],[42], plant-pathogenic [43], and intestinal pathogens [16]; suggesting that anaplerosis may be a common mechanism of microbial pathogenesis.

**Figure 7:**
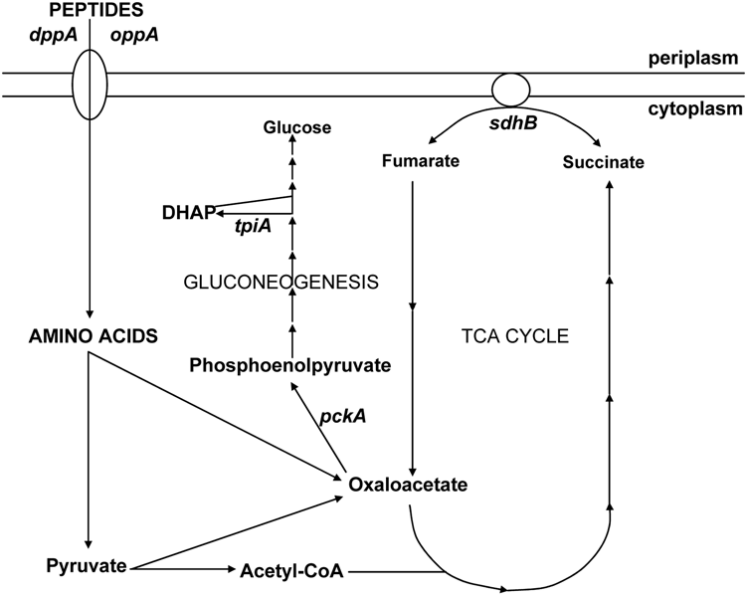
UPEC acquires amino acids and requires gluconeogenesis and the TCA cycle for fitness *in vivo*. Peptide substrate-binding protein genes *dppA* and *oppA* are required to import di- and oligopeptides into the cytoplasm from the periplasm. Short peptides are degraded into amino acids in the cytoplasm and converted into pyruvate and oxaloacetate. Pyruvate is converted into acetyl-CoA and enters the TCA cycle to replenish intermediates and generate oxaloacetate. Oxaloacetate is converted to phosphoenolpyruvate by the *pckA* gene product during gluconeogenesis. Mutations in the indicated genes *dppA*, *oppA*, *pckA*, *sdhB*, and *tpiA* demonstrated fitness defects *in vivo*.

This study comprehensively examines the role of pathogenic *E. coli* central metabolism in a disease model and provides insight not only into UPEC metabolism *in vivo* but also information regarding the nutrients available to support the growth of *E. coli* within the urinary tract. The proteomics experiments did reveal that UPEC growing in human urine induces expression of multiple isoforms of both dipeptide- and oligopeptide-binding proteins, both of which were found to be required for UPEC to effectively colonize the urinary tract. This indicates that these bacteria actively import short peptides in urine and this function may indicate that peptides are an important carbon source *in vivo*. Consistent with this, only bacteria with defects in peptide transport, gluconeogenesis, or the TCA cycle demonstrated a significant reduction in fitness *in vivo* in both the bladder and kidneys. These findings suggest a model that describes the biochemistry of *E. coli* during UTI. For optimal growth during infection, short peptides are taken up by UPEC and degraded into amino acids that are catabolized and used in a series of anaplerotic reactions that replenish TCA cycle intermediates and generate gluconeogenesis substrates (Fig. 7).

Certain glycolytic steps are irreversible and the reverse gluconeogenic reaction is performed by an enzyme specific for gluconeogenesis. Carbon flux through glycolysis and gluconeogenesis must be carefully controlled by the cell to avoid a futile cycle of carbon metabolism [44]. Allosteric regulation of enzymes that catalyze irreversible reactions in these pathways and catabolite repression are mechanisms used to avoid the futile cycle [45],[46]. A gluconeogenic-specific enzyme subject to allosteric regulation is phophoenolpyruvate carboxykinase that converts oxaloacetate to phosphoenolpyruvate [47]. Deletion of the gene *pckA* that encodes this enzyme resulted in a significant reduction in UPEC fitness *in vivo*. Because bacteria prevent glycolysis and gluconeogenesis from occurring simultaneously and deletion of *pckA* reduced fitness *in vivo*, we reason that carbon flux through gluconeogenesis during UPEC infection may be an important indication of amino acid catabolism *in vivo*.

It is not surprising that, in addition to gluconeogenesis, the TCA cycle is also required for UPEC fitness *in vivo*. These two pathways are connected and collectively described as “filling in” or anaplerotic reactions. The TCA cycle is necessary to provide substrates for gluconeogenesis when cells use amino acids as a carbon source. Gluconeogenic amino acids can be degraded to oxaloacetate or to pyruvate that can be converted to acetyl-CoA and enter the TCA cycle [47]. Oxaloacetate, a TCA cycle intermediate, is converted to phophoenolpyruvate during gluconeogenesis by PckA as described above. A mutation in the TCA cycle enzyme succinate dehydrogenase, *sdhB*, results in a UPEC strain that has reduced fitness *in vivo*. This finding suggests that UPEC are growing aerobically in the urinary tract because succinate dehydrogenase is replaced by fumarate reductase during anaerobic growth and therefore, future work could confirm if the reductive TCA cycle is not operating during UPEC infection. The requirement for peptide import and the TCA cycle for UPEC fitness during infection is consistent with the hypothesis that acetyl-CoA production from the degradation of amino acids could be occurring *in vivo* as has been shown by another group [48].

Interestingly, with the exception of peptide-transport proteins, up-regulation of protein expression in urine *ex vivo* did not correlate with functional importance *in vivo*. This could be due to the fact that many central metabolism genes are constitutively expressed and that human urine only partially mimics the complex lifestyle of UPEC during UTI [49]. The absence of host cells and the immune response during growth in urine *ex vivo* could in part account for this discrepancy. It also remains possible that mutants that lack growth defects in urine but demonstrate reduced fitness *in vivo* could represent genes or metabolic pathways that are required for intracellular phases of growth during cystitis [50].

Despite these disadvantages, up-regulation of both DppA and OppA expression was seen in urine and loss of either *dppA* or *oppA* was found to negatively impact UPEC colonization *in vivo*. Induction of *dppA* has been reported in a hypervirulent UPEC strain that has a lacks a functional D-serine deaminase gene (*dsdA*) [51]. Deletion of *dppA* in this mutant strain resulted in a loss of the hypervirulent phenotype *in vivo* and significantly reduced its ability to colonize the urinary tract in competition with wild-type [51]. Surprisingly, in contrast to our findings, this group found that mutation of *dppA* alone had no effect on UPEC fitness *in vivo* [51]. Due to lack of complementation, it is unclear from that work why loss of *dppA* dramatically attenuated a hypervirulent strain but had no effect on wild-type. Despite this inconsistency in that work, the importance of peptide transport for UPEC fitness *in vivo* is supported by the findings that loss of either *dppA* or *oppA* significantly reduced colonization of the urinary tract and that the reduced bacterial colonization in the Δ*dppA* strain can be restored to wild-type levels by complementing the mutant with a wild-type *dppA* gene.

In summary, defects in the both branches of the pentose phosphate pathway, the Entner-Doudoroff pathway, and glycolysis had limited or no impact on UPEC fitness *in vivo*. On the other hand, the TCA cycle- and gluconeogenesis-defective strains demonstrate significant fitness reductions during UTI. The utilization of short peptides and amino acids as a carbon source during bacterial infection of the urinary tract is supported by the observation that UPEC mutants defective in peptide import have reduced fitness *in vivo* while auxotrophic strains do not. Together, these findings provide compelling evidence to support the notion that catabolism of amino acids to form TCA cycle intermediates and gluconeogenic substrates is important for the ability of UPEC to infect the urinary tract efficiently. This shows that anaplerotic and central metabolism pathways are required for UPEC fitness *in vivo* and suggest microbial metabolism should be considered important for bacterial pathogenesis.

## Materials and Methods

### Bacteria and growth conditions

Strains were derived from *E. coli* strain CFT073, a prototypic UPEC strain isolated from the blood and urine of a patient with acute pyelonephritis [52]; its genome has been sequenced and fully annotated [53]. Isolated colonies were used to inoculate overnight Luria-Bertani (LB) cultures. Bacteria from overnight cultures were collected by centrifugation, washed with sterile PBS, and 10^6^ CFU were used to inoculate pre-warmed LB or human urine. To mimic iron-limitation in urine, LB containing 10 mM deferoxamine mesylate (Sigma) was used as a growth medium for comparative proteomics. For human urine cultures, mid-stream urine was collected into sterile sample containers from 8–10 male and female donors, pooled, and sterilized by vacuum filtration through a 0.22 µm pore filter. MOPS defined medium containing 0.2% glucose [54] with and without 10 mM L-arginine, L-serine, glycine, aspartatic acid, or D-serine (Sigma) was also used to test growth of mutant strains. Growth curves were established in triplicate using a Bioscreen bioanalyzer in 0.4 ml volumes; OD_600_ was recorded every 15 min. All cultures were incubated at 37°C; LB overnight and MOPS cultures were incubated with aeration; urine cultures were incubated statically. For preparation of proteins, UPEC isolate CFT073 was grown statically to exponential phase (OD_600_ = 0.25) in pre-warmed LB or human urine at 37°C in 5×100 ml cultures for each growth medium.

### Preparation of cytoplasmic proteins

Bacteria were harvested from 500 ml of culture by centrifugation (10,000× *g*, 30 min, 4°C) and lysed in a French pressure cell at 20,000 psi. Harvested cells were washed and resuspended in 10 ml of 10 mM HEPES, pH 7.0 containing 100 U of Benzonase (Sigma). Following two passes through the chilled pressure cell, lysates were centrifuged (7500× *g*, 10 min, 4°C) to remove unbroken cells and supernatants were ultracentrifuged (120,000× *g*, 1 h, 4°C) to remove membranes and insoluble material. Soluble proteins were quantified using the 2D Quant Kit (GE Healthcare) following the manufacturer's protocol and either used immediately in DIGE-labeling procedures or stored at −80°C.

### 2D-DIGE and MS/MS

For fluorescence difference in gel electrophoresis (2D-DIGE) [55], bacterial proteins were minimally labeled with cyanine-derived fluors (CyDyes) containing an NHS ester-reactive group as recommended by the manufacturer (GE Healthcare). To determine quantitative differences within the UPEC soluble proteome during growth in human urine, cytoplasmic proteins prepared from human urine cultures were labeled with Cy3, from LB broth with Cy5, and a pooled internal standard representing equal amounts of both urine and LB preparations with Cy2 as described previously [23]. Briefly, 50 µg of protein was incubated with 400 pmol CyDye for 30 min and the reaction was stopped by added 10 mM lysine. Following labeling, samples labeled with each CyDye were pooled (150 µg total protein), mixed with an equal volume of 2× DIGE sample buffer; 7 M urea, 2 M thiourea, 10 mM tributylphosphine (TBP) (Sigma), 2× biolytes 3–10 (Bio-Rad), 2% ASB-14 and incubated on ice for 10 min. For rehydration, samples were brought to 0.35 ml with 1× DIGE rehydration buffer (7 M urea, 2 M thiourea, 5 mM TBP, 1× biolytes 3–10, 1% ASB-14) and used to passively rehydrate pH 4–7 IPG strips (Bio-Rad) overnight at room temperature. Rehydrated IPG strips were equilibrated and subjected to isoelectric focusing for 50,000 V·h and second dimension SDS-PAGE on 10% gels within low fluorescence glass plates (Jule Biotechnologies, Inc.) and were run at a constant current of 55 mA at 4°C for 4 hr. Following SDS-PAGE, image acquisition and pixel intensity was obtained using a Typhoon scanner (GE Healthcare) and differential in-gel analysis and biological analysis of variance were performed using the DeCyder 6.5 software suite (GE Healthcare). Using this software, the normalized spot volume ratios from Cy3 or Cy5 labeled spots were quantified relative to the Cy2-labeled internal standard from the same gel. The Cy2-labeled standard was then used to standardize and compare normalized volume ratios between the Cy3 and Cy5 labeled proteins between gels representing three independent experiments to generate statistical confidence for abundance changes using student's *t*-test and ANOVA. To identify the proteins, 500 µg of cytoplasmic proteins were focused as described above and spots of interest were excised from a colloidal Coomassie-stained 2D SDS-PAGE gel and subjected to enzymatic digestion with trypsin. Mass spectra were acquired on an Applied Biosystems 4700 Proteomics Analyzer (TOF/TOF). MS spectra were acquired from 800–3500 Da and the eight most intense peaks in each MS spectrum were selected for MS/MS analysis. Peptide identifications were obtained using GPS Explorer (v3.0, Applied Biosystems), which utilizes the MASCOT search engine. Each MS/MS spectrum was searched against NCBInr. Tryptic digestion and tandem mass spectrometry were performed at the University of Michigan Proteome Consortium.

### Construction of UPEC metabolism mutants

Deletion mutants were generated using the lambda red recombinase system [56]. Primers homologous to sequences within the 5′ and 3′ ends of the target genes were designed and used to replace target genes with a nonpolar kanamycin resistance cassette derived from the template plasmid pKD4 [56]. Kanamycin (25 µg/ml) was used for selection of all mutant strains. Gene deletions begin with the start codon and end with the stop codon for each gene. To determine whether the kanamycin resistance cassette recombined within the target gene site, primers that flank the target gene sequence were designed and used for PCR. After amplification, each PCR product was compared to wild-type PCR product and in cases where size-differences are negligible; PCR products were digested with the restriction enzyme *Eag*I (New England Biolabs). Both the PCR products and restriction digests were visualized on a 0.8% agarose gel stained with ethidium bromide. For *in vivo* complementation, the *dppA* and *pckA* genes were amplified from CFT073 genomic DNA using Easy-A high-fidelity polymerase (Stratagene) and independently cloned into pGEN-MCS [28],[57] using appropriate restriction enzymes. The sequences of pGEN-*dppA* and pGEN-*pckA* were verified by DNA sequence analysis prior to electroporation into CFT073 Δ*dppA* or Δ*pckA* mutant strains.

### Experimental UTI

Six-to eight-week-old female CBA/J mice (20 to 22 g; Jackson Laboratories) were anesthetized with ketamine/xylazine and inoculated transurethrally over a 30 sec period with a 50 µl bacterial suspension per mouse using a sterile polyethylene catheter (I.D. 0.28 mm×O.D. 0.61 mm) connected to an infusion pump (Harvard Apparatus). To measure relative fitness, overnight LB cultures for CFT073 and the mutant strain were collected by centrifugation and resuspended in sterile PBS, mixed 1∶1 and adjusted to deliver 2×10^8^ CFU per mouse. Dilutions of each inoculum were spiral plated onto LB with and without kanamycin using an Autoplate 4000 (Spiral Biotech) to determine the input CFU/mL. After 48 hpi, mice were sacrificed by overdose with isoflurane and the bladder and kidneys were aseptically removed, weighed, and homogenized in sterile culture tubes containing 3 ml of PBS using an OMNI mechanical homogenizer (OMNI International). Appropriate dilutions of the homogenized tissue were then spiral plated onto duplicate LB plates with and without kanamycin to determine the output CFU/g of tissue. Plate counts obtained on kanamycin were subtracted from those on plates lacking antibiotic to determine the number of wild-type bacteria. Competitive indices were calculated by dividing the ratio of wild-type to mutant at 48 hpi by the ratio of wild-type to mutant input CFU/mL. Groups of 5 mice per co-challenge were used to determine defects in fitness, when a defect was apparent the co-challenge was repeated two more times with groups of 5 mice. Statistically significant differences in colonization (*P*-value<0.05) were determined using a two-tailed Wilcoxon matched pairs test. All animal protocols were approved by the University Committee on Use and Care of Animals at the University of Michigan Medical School.
